# Supplementary material for: Development of a computer-aided design software for the quantitative evaluation of aesthetic damage
Source: PLoS One. 2019 Dec 18;14(12):e0226322. doi: 10.1371/journal.pone.0226322 (PMC6919621; doi:10.1371/journal.pone.0226322)
Supplement: S1 Code site — 1. (ZIP) [file pone.0226322.s001.zip › [EESC_jr][Projeto]Relatorio_07_21/reports/templates_zip/gentelella-master/production/form.html]

Gentellela Alela! | 


Gentellela Alela!

Welcome,

## John Doe

  


### General

- Home 
  - Dashboard
  - Dashboard2
  - Dashboard3
- Forms 
  - General Form
  - Advanced Components
  - Form Validation
  - Form Wizard
  - Form Upload
  - Form Buttons
- UI Elements 
  - General Elements
  - Media Gallery
  - Typography
  - Icons
  - Glyphicons
  - Widgets
  - Invoice
  - Inbox
  - Calendar
- Tables 
  - Tables
  - Table Dynamic
- Data Presentation 
  - Chart JS
  - Chart JS2
  - Moris JS
  - ECharts
  - Other Charts
- Layouts 
  - Fixed Sidebar
  - Fixed Footer

### Live On

- Additional Pages 
  - E-commerce
  - Projects
  - Project Detail
  - Contacts
  - Profile
- Extras 
  - 403 Error
  - 404 Error
  - 500 Error
  - Plain Page
  - Login Page
  - Pricing Tables
- Multilevel Menu 
  - Level One- Level One
      - Level Two
      - Level Two
      - Level Two
    - Level One
- Landing Page Coming Soon

- John Doe
  - Profile
  - 50%
    Settings
  - Help
  - Log Out
- 6
  - John Smith
    3 mins ago

    Film festivals used to be do-or-die moments for movie makers. They were where...
  - John Smith
    3 mins ago

    Film festivals used to be do-or-die moments for movie makers. They were where...
  - John Smith
    3 mins ago

    Film festivals used to be do-or-die moments for movie makers. They were where...
  - John Smith
    3 mins ago

    Film festivals used to be do-or-die moments for movie makers. They were where...
  - **See All Alerts**


### Form Elements

Go!

## Form Design different form elements

- - Settings 1
  - Settings 2

First Name \*

Last Name \*

Middle Name / Initial

Gender

Male  

 Female

Date Of Birth \*

Cancel
Submit

## Form Design different form elements

- - Settings 1
  - Settings 2

Default Input

Disabled Input

Read-Only Input

Date Of Birth \*

Cancel
Submit

## Star Rating

- - Settings 1
  - Settings 2

#### Star Ratings Hover and click on a star

You gave a rating of 0 star(s)

Also you can give a default rating by adding attribute data-rating

You gave a rating of 4 star(s)

## Registration Form Click to validate

- - Settings 1
  - Settings 2

Full Name \* :

Email \* :

Gender \*:

M:
 F:

Hobbies (2 minimum):

Skiing
  
 Running
  
 Eating
  
 Sleeping
  

Heard us by \*:

Choose..
Press
Internet
Word of mouth
Message (20 chars min, 100 max) :

  
Validate form

## Form Basic Elements different form elements

- - Settings 1
  - Settings 2

Default Input

Disabled Input

Read-Only Input

Date Of Birth \*

Password

AutoComplete

Select

Choose option
Option one
Option two
Option three
Option four

Select Custom

Alaska
Hawaii
California
Nevada
Oregon
Washington
Arizona
Colorado
Idaho
Montana
Nebraska
New Mexico
North Dakota
Utah
Wyoming
Arkansas
Illinois
Iowa
Kansas
Kentucky
Louisiana
Minnesota
Mississippi
Missouri
Oklahoma
South Dakota
Texas

Select Grouped

Alaska
Hawaii

California
Nevada
Oregon
Washington

Arizona
Colorado
Idaho
Montana
Nebraska
New Mexico
North Dakota
Utah
Wyoming

Alabama
Arkansas
Illinois
Iowa
Kansas
Kentucky
Louisiana
Minnesota
Mississippi
Missouri
Oklahoma
South Dakota
Texas
Tennessee
Wisconsin

Connecticut
Delaware
Florida
Georgia
Indiana
Maine
Maryland
Massachusetts
Michigan
New Hampshire
New Jersey
New York
North Carolina
Ohio
Pennsylvania
Rhode Island
South Carolina
Vermont
Virginia
West Virginia

Select Multiple

Choose option
Option one
Option two
Option three
Option four
Option five
Option six

Input Tags

Checkboxes and radios
  
Normal Bootstrap elements

Option one. select more than one options

Option two. select more than one options

Option one. only select one option

Option two. only select one option

Checkboxes and radios
  
Normal Bootstrap elements

Checked

Unchecked

Disabled

Disabled & checked

Checked

Unchecked

Disabled

Disabled & Checked

Switch

Checked

Unchecked

Disabled

Disabled Checked

Cancel
Submit

## Form Buttons Sessions

- - Settings 1
  - Settings 2

Button addons

Go!

Go!

Button addons

Action 

- Action
- Another action
- Something else here
- Separated link

Go!

## Text areasSessions

- - Settings 1
  - Settings 2

- Huge
- Normal
- Small

Add

## Form Input Grid form input

- - Settings 1
  - Settings 2

## Form Design different form elements

- - Settings 1
  - Settings 2


Gentelella - Bootstrap Admin Template by Colorlib
